# Laughter therapy as an intervention to promote psychological well-being of volunteer community care workers working with HIV-affected families

**DOI:** 10.1080/17290376.2017.1402696

**Published:** 2017-11-23

**Authors:** Irene Hatzipapas, Maretha J. Visser, Estie Janse van Rensburg

**Affiliations:** ^a^ MA Counselling Psychology, Department of Psychology, University of Pretoria, Pretoria, South Africa; ^b^ PhD, Professor in the Department of Psychology, University of Pretoria, Pretoria, South Africa; ^c^ PhD, Lecturer in the Department of Psychology, University of Pretoria, Pretoria, South Africa

**Keywords:** laughter therapy, volunteer community care workers, orphans and vulnerable children, psychological well-being, mixed methods

## Abstract

The study explores the experiences of volunteer community care workers working with HIV-affected families, participating in laughter therapy. Laughter therapy is being used as an intervention to positively influence individuals experiencing various forms of emotional distress. Community care workers play a vital role in the support of the HIV/AIDS-infected and -affected members in communities. The nature of this type of work and their limited training contributes to high levels of secondary trauma and emotional exhaustion. The purpose of the study was firstly, to explore the effects of working with orphans and vulnerable children (OVC) on the community care workers and secondly, to establish the impact that laughter therapy has to positively combat stresses of working within the care workers’ environment. All the community care workers from a community-based organisation that provides care for HIV/AIDS-infected and -affected OVC and their families in the greater region of Soweto, South Africa, took part in daily laughter therapy sessions for one month. To assess the experiences of participants of laughter therapy, seven community care workers agreed to participate in a mixed method assessment. Interviews were conducted before and after the intervention using the Interpretative Phenomenological Analysis as framework. As supportive data, a stress and anxiety and depression scale were added in the interview. Participants reported more positive emotions, positive coping, improved interpersonal relationships and improvement in their care work after exposure to laughter therapy. Quantitative results on stress, anxiety and depression for each participant confirmed observed changes. Laughter therapy as a self-care technique has potential as a low-cost intervention strategy to reduce stress and counteract negative emotions among people working in highly emotional environments.

## Introduction

A large number of children who have been orphaned or affected by HIV/AIDS in South African are cared for by either their immediate or extended families, specifically grandparents or older siblings (Foster, Levine, & Williamson, 2005; Schenk et al., 2008; Townsend & Dawes, 2009). To address some of the challenges faced by households that care for orphaned and vulnerable children (OVC) (Vermaak, Mavimbela, Chege, & Esu-Williams, 2004), various community-based organisations (CBOs) offer care services to these HIV-affected families (Schenk, Michaelis, Sapiano, Brown, & Weiss, 2010; U.S. President’s Emergency Plan for AIDS Relief, 2012). Because of limited professional services, various different categories of care workers developed (Cameron, Coetzee, & Ngidi, 2009) as part of task shifting in health and social services (Callaghan, Ford, & Schneider, 2010; Schneider & Lehmann, 2010; WHO, 2007). Community care workers are community members with no formal professional or paraprofessional qualifications that are trained to provide specific healthcare or social services (Cameron et al., 2009; Lewin et al., 2005). CBOs recruit volunteering community members, provide in-service training, support and a stipend for them to take care of various practical, social and psychological needs of a few HIV-affected families in their community (Uys, 2002; Uys & Cameron, 2003).

The positive impact of doing community care work within the HIV/AIDS context for the community and the volunteer community care workers has been well documented (for example, Akintola, 2008; 2010; Wringe, Cataldo, Stevenson, & Fakoya, 2010). Providing care to people in need is often regarded as a very rewarding and altruistic action. However, stress and depression have also been reported as possible negative effects on community care workers (Armstrong, 2000; UNAIDS, 2000). Care workers are often exposed to illness and death, hardships and trauma of clients, emotional involvement and high expectations and responsibilities, often in a context of low remuneration and lack of recognition (Akintola, 2008; Cataldo, Kielmann, Kielmann, Mburu, & Musheke, 2015; Peltzer & Davids, 2011). Exposure to intense emotional contexts can result in secondary traumatisation (being indirectly traumatised by helping others) which could lead to compassion fatigue in professionals (Berger, Polivka, Smoot, & Owens, 2015; Coetzee & Klopper, 2010; Meadors & Lamson, 2008). Because of limited training care workers are often over-involved with the problems of their clients and are even more at risk of experiencing secondary traumatisation and emotional exhaustion (Pirraglia et al., 2005; Shapiro, Brown, & Biegel, 2007; Visser & Mabota, 2015). Peltzer and Davids (2011) found that 78% of care workers (lay counsellors) in their study experienced high levels of job stress and that 31% felt emotionally drained by their work. These care workers are often also burdened by their own personal needs which could negatively influence the quality of their care services (Cataldo et al., 2015; Coetzee & Klopper, 2010; O’Neill & McKinney, 2003).

The psychological well-being of care workers is important as it directly influences their ability to provide effective services to the families in their care (Corey, 2013). Van Dyk (2013, p. 286) states that ‘ … it is important for the self-preservation of care workers and for their emotional survival that they should take care of themselves’. Self-care, the cornerstone of compassion fatigue prevention, is often neglected by care workers (Geteri & Angogo, 2013; Wentzel & Brysiewicz, 2014). Cameron et al. (2009) therefore advocate the provision of interventions to assist community care workers to deal with their emotional needs and job stress. Research shows that taking care of the providers on a personal level had positive impact on their stress and compassion fatigue levels (Meadors & Lamson, 2008). As an effort to promote self-care of care workers, this study aims to explore the benefits of laughter as a therapeutic intervention to increase the psychological well-being of community care workers.

### Laughter as an intervention to promote well-being

Laughter is a familiar action for most individuals. It has been the subject of consideration by a long and honourable list of thinkers, from the Greek philosophers (Plato, Aristotle and Hobbes) to modern psychologists. However, there is little academic literature and research of laughter and the therapeutic use of laughter to enhance psychological well-being in the human and social sciences. A number of theories have attempted to explain the value of laughter. Kant and Schopenhauer regarded laughter as a reaction to perceived incongruity, while the Relief Theory is based on the premise that laughter is the release of surplus nervous energy (Morreall, 1983). Furthermore, Morreall (1986) theorised that humour and laughter can be a path to mental health as it may provide an individual with some form of relief from the mundane aspects of human existence. Laughter can therefore be associated with the framework of positive psychology.

Positive psychology aligns its focus with mental health and building positive qualities, rather than mental illness (Donaldson, Dollwet, & Rao, 2015; Snyder & Lopez, 2002). The essence of Positive Psychology is captured in the following statement:

Psychology is not just the study of disease, weakness, and damage; it also is the study of strength and virtue. Treatment is not just fixing what is wrong; it also is building what is right. Psychology is not just about illness or health; it is about work, education, insight, love, growth, and play. (Seligman, 2002, p. 4)

In line with this way of thinking, building an individual's strengths is possibly the most effective and powerful therapeutic intervention (Seligman, 2002).

The ability to laugh assists individuals to develop an affinity towards positive emotions, and the expression thereof, which in turn also has a positive influence on their affect (Junkins, 1999). Fredrickson (2001) claims positive emotions contributes to personal well-being. This occurs by broadening thought-action or expanding the individual's attention and ideas. It helps in undoing the effects of negative emotions and increasing psychological resilience and personal resources. Furthermore, positive affective experiences contribute to and have a long lasting effect on personal growth and development (Fredrickson, 2001).

Although laughter can be associated with silliness and frivolousness by some, laughter therapy is based on the premise that laughter is a primary cathartic trigger, enabling the release of previously unexpressed emotions (Junkins, 1999). Cousins (1979) was one of the first authors who wrote about laughter as a therapeutic intervention based on his own experiences in overcoming a serious chronic disease. He subjected himself to continuous viewings of his favourite comedy shows. He advocated that 10 minutes of laughing gave him two hours of drug-free pain relief.

In addition, Berk et al. (1989) examined the effects of laughter on neuroendocrine hormones that are involved in classical stress responses. The researchers concluded that joyful laughter modifies or reduces some of the neuroendocrine hormone levels associated with stress. Similar results were found in an experiment where participants were exposed to a humorous video of their choice, compared to participants in the control group that viewed a tourism video. The research findings showed that the cortisol levels and self-reported stress levels of participants exposed to the humorous situation decreased more rapidly than those of the control group (Bennett, Zeller, Rosenberg, & McCann, 2003). Furthermore, laughter may not only buffer the effects of stress but may play an important role in enhancing the pleasures of positive life events (Martin, Kuiper, Olinger, & Dance, 1993). According to Colom, Alcover, Sanchez-Curto, and Zarate-Osuna (2011), laughter activates the subcortical regions with specific reference to the nucleus accumbens, a key component of the mesolimbic dopaminergic system. This reward system provides pleasure when something of value is obtained. In an earlier study, laughter was found to be utilised as one of the major cathartic practises for releasing or healing emotional pain. When people laugh, they are releasing painful feelings which could have been repressed over a long period of time (Goodheart, 1994).

There seems to be some obstacles to utilising laughter therapy. During the formative years, children are conditioned to laugh when it is socially appropriate. This conditioning negatively influences the application of laughter as a therapeutic intervention. Individuals tend to feel uncomfortable and fear losing control cathartically when exposed to laughter, crying or anger expressions. On the contrary, losing control of one's emotions cathartically allows one to regain control of one's life through flexible, creative and caring means (Goodheart, 1994). Despite the biological evidence that laughter therapy has a positive effect on distressful experiences of participants (Bennett et al., 2003; Colom et al., 2011), there is still scepticism among psychologists and psychiatrists about the value of laughter as a therapy for intense emotions such as depression and anxiety (Junkins, 1999). There are also questions about how participants experience this therapy and how it contributes to positive experiences. This study explored the experiences of community care workers that participated in laughter therapy.

### Laughter therapy

Given that laughter has been found to have several positive effects, many variations of laughter therapy have been developed. For the purpose of this study, Aerobic Laughter Therapy (ALT), a cognitive behavioural technique, was utilised.

According to the Association for Applied and Therapeutic Humor, therapeutic humour is defined as:

an intervention that promotes health and wellness by stimulating a playful discovery, expression or appreciation of the absurdity or incongruity of life's situations. This intervention may enhance health or be used as a complementary treatment to facilitate healing or coping, whether physical, emotional, cognitive, social or spiritual. (Association for Applied and Therapeutic Humor, 2000, para 5)

Based on this definition, ALT utilises techniques that promote playfulness and expression of the frustrations and stressors of life. ALT sessions typically begin with physical warm up activities, which include stretching, clapping and body movements. These techniques promote childlike playfulness to assist in breaking down inhibitions. Participants are then led through a series of breathing techniques, followed by numerous laughter exercises that combine acting and playful visualisation techniques (Kataria, 2002). These exercises, when combined with the strong social dynamics of group behaviour, can lead to prolonged and wholehearted unconditional laughter. ALT is delivered according to rigorous standardised procedures ensuring consistent delivery and results (Gee, 2011). The programme was developed during three years of practical trials to help individuals and groups to combat the effects of stress and depression (Gee, Jaffer, & Matanda, 2010; Jaffer, Gee, & Matanda, 2011). Currently, laughter therapy, specifically ALT, is implemented by a non-profit organisation InHappiness (International Happiness Institute) in palliative and home-based care settings in South Africa to counter stress and depression and builds health and happiness in these target groups (http://www.inhappiness.org/about-inhappiness.htm).

## Aim of the study

The aims of the research were (1) to understand the emotional experiences of community care workers taking care of HIV-affected families and (2) to explore their experiences of laughter therapy sessions as a form of self-care to determine the value of laughter sessions in this context.

## Method

### Context of the research

The research was done among the volunteer community care workers of a CBO that provides an integrated child/family centred programme for OVC in Soweto, South Africa. The CBO provides home visits that include psycho-social, educational, nutritional and household support for over 300 children and their families. The CBO has been in operation for over 7 years. The CBO recruits community members as care workers and provides them with basic in-service training and support. The specific services the care workers provide for HIV-affected families include: distribution of food parcels, access to social grants, community education programmes, home visits and palliative care, assistance with household chores, limited counselling and identification of needs and referral to relevant services.

InHappiness Institution, a non-profit organisation sponsored by UNAID, offered laughter therapy sessions for the care workers of the CBO in the form of self-care activities. The authors were granted permission to conduct the research during a series of laughter therapy sessions in this context.

### Research design

A mixed methods research design involves combining quantitative and qualitative research techniques, methods, approaches and concepts into one single study (Johnson & Onwuegbuzie, 2004). The rationale for this type of research is that using both qualitative and quantitative research methods provides a better understanding of a research problem and more validity of the findings than using either research approach in isolation (Creswell, 2009; Johnson & Onwuegbuzie, 2004). It also draws on the strengths of each approach while giving a more holistic and complete understanding of the phenomenon under exploration. In this study, the qualitative data analysis is given priority and quantitative results are used to support the qualitative results – it is thus a qualitative dominant mixed analysis (Johnson & Onwuegbuzie, 2004).

The qualitative study made use of the Interpretive Phenomenological Analysis (IPA) (Smith & Osborn, 2008). The aim of this approach is to understand how people attempt to give meaning to their personal and social worlds (Smith & Osborn, 2008). Edmund Husserl, a key developer of phenomenology, describes the aim of this method as to clarify how a certain phenomenon is experienced and presents itself to human awareness (Spinelli, 2005). This approach allows the researcher to explore and understand the personal world of another through a process of interpretation (Willig, 2008). In this study, the community care workers were guided towards communicating their experiences of being a care worker and participating in laughter therapy sessions.

The quantitative data consist of pre-and post-scores for each participant on two questionnaires to determine whether change took place. Because of the small sample size, each individual's scores could be interpreted and compared with his/her qualitative data. The mixed methods approach thus provides results using different data collection strategies.

### Participants

Qualitative and quantitative data were collected from a sample of care workers that was selected using purposive sampling (Teddlie & Yu, 2007). Purposive sampling is a method that selects individuals that meet particular criteria (Terre Blanche, Durrheim, & Painter, 2006). Participants were required to meet the following inclusion criteria:

- Being a community care workers at the CBO
- Will participated in the ALT programme provided by InHappiness
- Have an English proficiency comparative to a grade 12 qualification to enable the researcher to conduct interviews in English. (This was done so that the richness and meaning of language will not be lost in the process of translation.)

Participants were recruited through the director of the CBO, depending on their availability, suitability to the study and willingness to participate. From the 30 care workers at the CBO who participated in the laughter sessions at the time, 10 participants, 3 male and 7 female, were willing to participate in the research. At the time of the post-assessment, three of the care workers, one male and two females, were no longer employed by the CBO (due to high turnover) and thus dropped out of the study. The participants that completed both interviews were thus two males and five females between the ages of 20 and 38 (*M *= 28.83, SD = 7.44). They worked for the CBO for between 2 months and 7 years. Each of them had between 10 and 15 families in their care at any given time.

### Data collection

Data were collected through personal face-to-face semi-structured interviews and two short questionnaires before and after completion of the laughter therapy sessions.

#### Interviews

Through semi-structured interviews, the researcher created an environment of openness and trust wherein the participants were able to express their views openly. A question guide with open-ended questions permitted the participants to share their experiences using their own words. The goal of the pre-intervention interview was to explore the experiences of working as care workers and the impact care work had on the participant's psychological well-being. Examples of questions were: (1) How do you experience your work as a care worker? (2) How do you know when you are stressed? (3) How does stress affect your work with the children in your care? (4) How do you deal with upsetting problems?

In the post-intervention interviews, the researcher asked questions pertaining to the participants’ experience during and after the laughter sessions and the effect these sessions have had on their personal well-being. Example of questions: (1) Describe your experiences of the laughter sessions? (2) In what way has laughter influenced the way you view your work and your life in general?

The face-to-face interviews, before and after the therapy, were conducted by the researcher in the participants’ place of work and were approximately 30 minutes long. The interviews were tape recorded, with the permission of the participants.

#### Questionnaires

Two short questionnaires were administered before and after the laughter therapy intervention to provide additional data on the change in experiences of anxiety, depression and stress. Psychometric scales are often regarded as providing more rigorous data than qualitative data from interviews.

#### Hospital anxiety and depression scale (HADS)

The HADS (Zigmond & Snaith, 1983) was used to assess levels of anxiety and depression among care workers. The HADS was originally developed to screen for anxiety and depression among people attending a hospital as out-patients (Zigmond & Snaith, 1983). The scale has since been widely used as a reliable and valid measure to assess anxiety and depression in various populations (Bjelland, Dahl, Haug, & Neckelmann, 2001). The HADS consists of 14 items, divided into two subscales: anxiety and depression. Items that relate to anxiety include ‘I feel tense or wound up’ whereas items that relate to depression include ‘I have lost interest in my appearance.’ Response categories were on a 4-point scale of how often the respondent feels that way. According to Zigmond and Snaith (1983), scores of 11 or more on either subscale are indicative of depression and anxiety, where scores of 8–10 represent ‘borderline’ cases and 0–7 acceptable mental wellness. A study conducted by Mykletun, Stordal, and Dahl (2001) confirmed that both the anxiety and depression subscales were found to be internally consistent, with a Cronbach coefficient of 0.80 for anxiety and 0.76 for depression.

#### Perceived stress scale (PSS)

The PSS is a widely used psychological assessment of perception of stress (Cohen, Kamarck, & Mermelstein, 1983). The PSS is a 10-item scale that measures the degree to which particular events in one's life are considered stressful. High scores indicate more perceived stress. Items consist of questions such as ‘How often have you felt nervous or stressed?’ Participants rated how often they had experienced these feelings in the last month on a 5-point Likert scale ranging from 0 (*never*) to 4 (*very often*). The internal consistency was found to be 0.82 and test–retest reliability found to be 0.77 (Cohen et al., 1983; Remor, 2006). A Cronbach's alpha of 0.72 was obtained using a sample of South African adults (Hamad, Fernald, Karlan, & Zinman, 2008). The PSS was found to be a significant predictor of appraised stress levels for a heterogeneous community group (Cohen et al., 1983).

### Research process

In the pre-intervention interview, the researcher assisted each participant in completing the two questionnaires, whereafter the personal face-to-face semi-structured interview was conducted. Two weeks after the completion of the pre-intervention data collection, the participants were introduced to the trainers of the InHappiness laughter programme. The trainer was the founder of the Inhappiness Institute, a certified trainer (www.inhappiness.org). Participants were introduced to ALT through the education of laughter and numerous childlike techniques. The care workers participated in daily 10–15 minutes of group laughter sessions for a month. Sessions started with physical activity, breathing exercises and playful activities to break inhibitions down. This was followed by numerous laughter exercises that combined acting and playful visualisation techniques (Kataria, 2002). These exercises, when combined with the strong social dynamics of group behaviour, can lead to prolonged and wholehearted unconditional laughter.

The researcher conducted the post-intervention interviews after a period of one month of daily laughter sessions. The interviews may have had therapeutic value, because the participants shared their experiences and emotions openly.

### Data analysis

The contents of the tape recordings of the interviews were transcribed verbatim and analysed using the IPA methodology (Smith & Osborn, 2008). The researcher interpreted the experiences of the participants as they tried to make sense of what happened to them before, during and after the laughter therapy. This involved reading and re-reading the text to extrapolate themes which were then clustered according to higher order themes. Paraphrased extracts were used to make the voice of the participants heard. The researcher previously participated in laughter therapy sessions and was interested in how others experienced these sessions. To reduce bias, the researcher maintained reflexivity throughout the process through the recordings of her encounters in a journal. This ensured self-monitoring in order to focus on the participants’ experiences and not on the researchers’ assumptions and expectations. The researcher liaised with a colleague who has done an independent analysis of data to reach consensus on the main themes. Furthermore, the researcher discussed her interpretations with participants to validate her interpretations. This was done to enhance trustworthy interpretation of the data (Smith & Osborn, 2008).

The pre- and post-results of the scale scores of each participant were presented in the format of graphs. The graphs illustrated change that took place after the intervention. The data from the qualitative and quantitative analysis were triangulated to understand the reactions of participants.

### Ethical approval

The study was approved by the ethics committee of the Faculty of Humanities, University of Pretoria. Participation was voluntary and participants signed informed consent forms.

## Results

### Themes from pre-intervention interviews

**Fig. 1.**
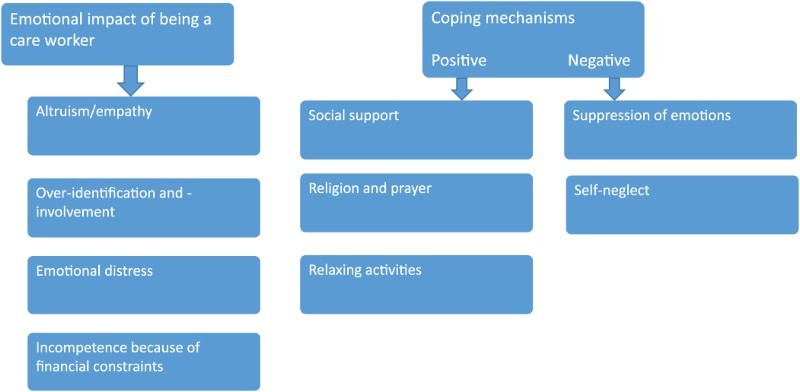
Themes and sub-themes on experiences of being an OVC care worker.

The themes that emerged from the data analysis were: emotional impact of being a care worker and coping mechanisms they used. Each main theme and sub-themes are illustrated in Fig. 1 and discussed below. To protect the identity of the participants, pseudonyms have been used in this study.

#### Emotional impact of being a care worker

##### Altruism/empathy

Most of the care workers voiced that they have a strong bond with the families in their care. They understand the challenges the children were experiencing and felt the responsibility to make a difference in the children's lives.

Being a care worker it's all about responsibility and loving and respecting other people. And putting yourself like in their shoes how they feel. (Gift, male, 29 years, 7 years of experience as a care worker)

The care workers’ understanding of the children is often built on their own experiences in similar situations. They thus identified strongly with the plight of the families. They wanted to help families because they received help themselves or did not receive help and wanted to help others to be better off than they were:

It's because of my growing up, you know seeing the challenges and like wanting to help other people. I would understand exactly where they’re coming from. (Precious, female, 38 years, 5 years of experience as a care worker)

When I became an orphan, I was cared for and looked after by the care workers in this CBO. Now I want to share the love that I was shown back to the community through my work as a care giver. (Mpumi, female, 20 years, 6 months of experience as a care worker)

I never had someone to be my care worker. My situation is almost their situation, so I decided to be a care worker so that I can inform them and help them grow and be strong. (Kenzo, male, 20 years, 2 years of experience as a care worker)

##### Over-identification and -involvement

Most of the care workers were themselves from families affected by HIV/AIDS. They thus understand them but often over identify with them and become overly involved. For example:

They are like my real family. Even the one weekend I just go and visit those families, I just took a long walk and see how they are doing. (Gift, male, 29 years, 7 years of experience as a care worker)

The care workers expressed that they get personally involved with the children. This often triggered their own unresolved emotional issues which results in sleepless nights and intense sadness. One caregiver said she feels ‘pains in my heart’ when listening to the hardship of children and their families.

Most of the time I cry about them. But I tell myself that there are things I cannot do even though I want to help the children. (Precious, female, 38 years, 5 years of experience as a care worker)

##### Emotional distress

In their daily work duties, the care workers experienced numerous challenges that contributed to their experience of negative emotions. Some of the participants reported intense sadness and physical symptoms of stress such as severe headaches, loss of appetite, fatigue and inability to focus on their work. The care workers thus experienced high levels of emotional exhaustion.

I have a lot of headaches and I sleep a lot. I don't have an appetite. I don't eat and I don't like noise. I’m short tempered and I cry most of the time. (Mpumi, female, 20 years, 6 months of experience as a care worker)

Sometimes I’m being stressed. I suffer from severe headaches. When I start thinking about the situation, my head would start to be sore. (Gift, male, 29 years, 7 years of experience as a care worker)

##### Incompetence because of financial constraints

Most of the participants reported the limited financial resources available at the CBO to meet the needs of the community, as a major stress factors. The care workers conveyed feelings of being stressed, exhausted, frustrated and at times overwhelmed especially because they feel unable to help families and children to address their needs, as illustrated below:

Why am I here? I feel so useless, because I can't help this person with money. They come to you thinking you’ll help but you can't help, you have no resources, your hands are tied. It drains me, it drains me, it puts me down. (Mpho, female, 32 years, 3 years of experience as a care worker)

It can become very difficult to look the person in the eyes and tell them I can't help you, go home. Even though you send them to other places, you know those places won't help. They come here because they saw you helping other people. If I had this (money) I would help. (Mpho, female, 32 years, 3 years of experience as a care worker)

#### Coping mechanisms

Given the challenges in their work environment, the care workers have adopted some positive and negative coping mechanisms. Some positive approaches were the following.

##### Social support

The care workers received formal forms of support from their management. In addition, they used informal support from their co-workers, family and friends. They described talking about their frustrations and fears as a way of coping with their daily stressors. It helped them to overcome the feelings of being overwhelmed by the burdens and limitations of their work environment. Support systems buffered their levels of distress:

Usually I talk it over with my colleagues. I can bounce it off the people that are strong. When you start saying that, they calm you down saying eh relax. Here at work I have this friend. She is my strength, when I feel weak and down, I go to her*.* (Mpho, female, 32 years, 3 years of experience as a care worker)

##### Religion and prayer

Many of the participants reported making use of their religious beliefs and affiliations in times of stress. They prayed, went to church and participated in other religious activities. These participants explained experiencing emotional relief and support through these pursuits:

Emotionally, you know, I grew up in this kind of family that always tell me that when you are kind of stressed, go to church. You know, I survived by that. (Thandi, female, 34 years, 3 years of experience as a care worker)

##### Relaxing activities

The participants utilised numerous kinds of relaxation activities such as reading, walking, creative arts and listening to music. They expressed feeling relieved during these activities as it allowed them to process their work circumstances. They gained a sense of rejuvenation through relaxation activities.

Negative coping strategies include the following:

##### Suppression of emotions

Most of the participants reported that in order for them to work effectively with the children, they had to put their own feelings aside. Their primary concern was the well-being of the children. However, most participants voiced that suppressing their feelings has had a negative impact on their own emotional well-being. For example:

When you come here, you have to snap out of it. You don't want to take your stressors and put it on them. You want them to feel happy. (Mpho, female, 32 years, 3 years of experience as a care worker)

At times I tell them that I might look like this strong person but inside I’m hurting and (crying) most of the time I do suppress my feelings and act as if nothing is happening, but I know deep inside it hurts. (Precious, female, 38 years, 5 years of experience as a care worker)

##### Self-neglect

All the participants noted that it was their primary concern to meet the needs of the children, often at the expense of their own well-being. In the long run, lack of self-care and emotional support result in feelings of incompetence and infective work:

When I am stressed of course I don't take good care of myself. Even my siblings will notice. I lose concentration on the things that I was supposed to do. I just end up forgetting them. (Thandi, female, 34 years, 3 years of experience as a care worker)

### Themes from post-intervention interviews

Care workers expressed that laughter therapy played an important role in their lives and that it made them feel well. The discussion of the intervention generated much laughter during the interviews with the care workers. They reported that laughter helped them to get through difficult situations in the work place, as well as in their personal lives. Laughter relieved tension and strengthened the interactions between the care workers.

Four themes emerged from the interviews conducted after the care workers participated in one month of daily group laughter therapy sessions.

#### Positive emotional experiences

Most of the participants were initially sceptic about the value of laughter therapy. However, as the sessions progressed they began to enjoy it. In contrast with the pre-interviews, they reported experiencing a variety of positive emotions such as joy, happiness, relief and hope. This is illustrated in the following quotes:

I feel I have joy in my heart after laughing, you feel relieved. Eish, I don't know how to explain it but you feel you are happy. (Victoria, female, 34 years, 6 years of experience)

I felt something like relief in my body, like when you feel weight on your shoulders, but then I felt relieved and now I feel so light. (Mpho, female, 32 years, 3 years of experience)

Immediately when you are laughing there is something that will move you from this situation you are in and go to another better situation. It gives you hope. (Gift, male, 29 years, 7 years of experience)

You know they were amazed to hear that with only laughter it can be able to change your life. (Precious, female, 38 years, 5 years of experience)

#### Positive coping

The participants reported that laughter had changed the way they interpreted situations. Laughter worked as an effective tool to help the participants see a negative incident in a positive light. For example:

There is stress out there, but you have to look at it in a different perspective and not always stress about things. Be level-headed not to go down with stress. (Mpho, female, 32 years, 3 years of experience)

I used to worry a lot but now it's different. Through the laughter sessions it's better because now I know how to control myself towards anger, towards bad emotions, thinking a lot. Yes it has helped a lot. (Thandi, female, 34 years, 3 years of experience)

Participants were given the daily opportunity to express and release the emotions that they had ignored for so long. They therefore expressed suppressed emotions and felt a sense of relief.

Through doing the laughter sessions in a group, the care workers developed support from co-workers as an effective coping mechanism in a stressful work environment.

#### Improved interpersonal relationships

For the care workers, laughter functioned as a binding factor in relationships. They felt that laughing as a group had strengthened their work relationships and improved their relationships with friends and family as well. It awakened them to want to be more sociable and interactive with others:

You know, before I just need to be alone, but now I know to call them, let's sit together and I’m engaging much more with them than before. So you find through laughter you are able to engage with other people. (Thandi, female, 34 years, 3 years of experience)

Whenever somebody says something it doesn't bother me anymore, I don't take it personally. Those people that I pushed away from me, I need them to be around. (Precious, female, 38 years, 5 years of experience)

I was this person that was not in the mood to be with people. But since after (the laughter) I just really wanted to be with people, wanted to see other people. (Precious, female, 38 years, 5 years of experience)

Positive social interactions served to reinforce their positive emotions, thereby creating a sustainable system of wellness.

#### Improvement in care work

Care workers had more positive feelings, were more hopeful and made intimate contact with the children in their care. Some participants used laughter in their contact with the children to bring hope in difficult situations:

I feel lighter. I give them hope. You laugh when you touch somebody and some kids have never been touched before. You touch them and they smile because laughter brings a smile to the face. Your own face opens up, like your eyes and everything when you are laughing. And then you give the next person hope, they will get through it somehow. There is hope even if they are in a bad situation. (Mpho, female, 32 years, 3 years of experience)

Care workers realised that they have to change themselves before they can expect others to change. Most of the participants indicated some changes in their behaviours and in the way they view their situations:

If you want people to change, you have to change first. By changing the way I look at things I will be impacting others. (Mpho, female, 32 years, 3 years of experience)

### Integration with quantitative results

**Fig. 2.**
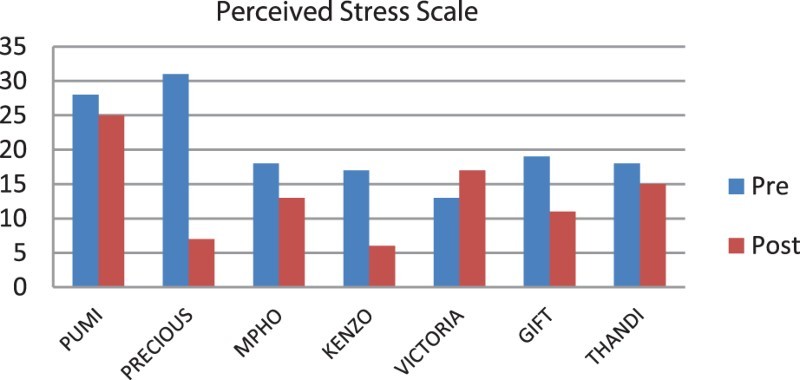
Pre- and post-intervention stress scores per participant.

**Fig. 3.**
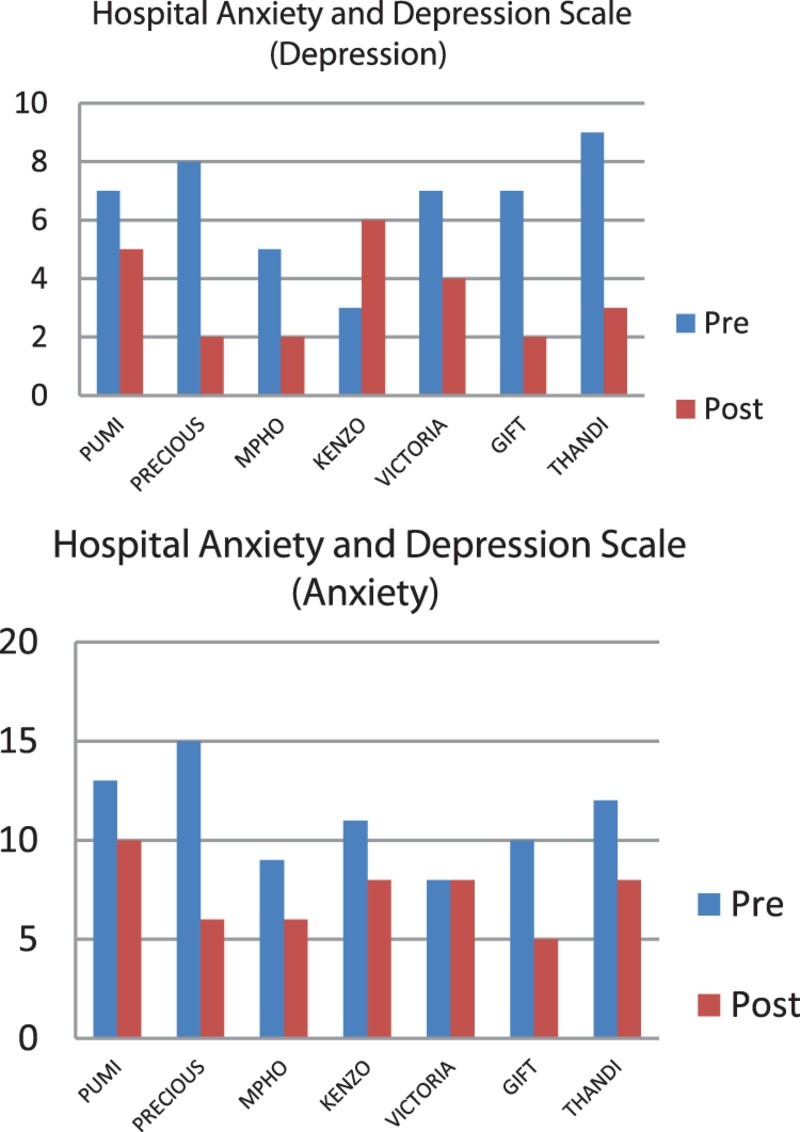
Pre- and post-intervention test analysis (HADS).

Two questionnaires were administered before and after the laughter therapy intervention to identify changes that took place with regard to stress, depression and anxiety of each participant. The scores of each participant were plotted in Figs. 2 and 3 to understand the participants’ reactions.

The scores for each participant showed some interesting results. Although most of the participants experienced less stress, anxiety and depression after the therapy sessions, all participants did not have similar experiences. For example, Victoria reported more stress after the intervention, her levels of anxiety remained the same, while her experiences of depression were lower after the intervention. Victoria experienced her work as frustrating because of the minimal salary she receives and limited financial resources available to care for the children. During the intervention period, she had applied for a learnership position to improve her circumstances. Her elevated levels of stress could be contributed to her uncertainty of her capabilities to get the learnership.

Thandi experienced the highest level of depression before the intervention. Since her mother passed away, she was left to take care of her siblings. The intervention helped her to process her feelings and she reported significantly less depression after the intervention. Similarly, Precious appeared to experience the largest differences between pre- and post-intervention assessments on all three scales. Before the intervention, she was depressed and withdrew to the extent of attempting suicide earlier in the year. In the post-intervention interview, she displayed energy and optimism, more confidence and was more sociable. She describes her reaction to laughter therapy as follows: ‘I think I am loving myself more and understand that it's up to me to want to be happy.’

## Discussion

The purpose of the study was to explore the emotional experiences of community care workers attending to HIV-affected families. Secondly, their experiences of laughter therapy sessions as an intervention strategy to promote their psychological well-being were explored.

The nature of their work involves a mammoth task to care for the infected and affected families. Because care workers were from the same community and many of them were from HIV-affected families themselves, they could express empathy and altruism to help others in similar situations. They could thus identify with the plight of the community in which they work, which contributed to involvement and job satisfaction (Van Dick, Van Knippenberg, Kerchreiter, Hertel, & Wieseke, 2008). Though, over-identification and over-involvement could result in emotional distress (Collins, 2007). Their lack of formal training can contribute to the experience of secondary trauma, following from being confronted daily with the traumas of their clients. It seemed that some of the care workers experienced high levels of emotional exhaustion, especially when they did not have the resources to address the families’ needs (Price et al., 2013; Storey & Billingham, 2001).

In order to buffer the effects of emotional distress, care workers used positive and negative coping mechanisms. In order for them to carry out their work effectively, they had a tendency of masking, overlooking and ignoring their own emotions. They often neglected their own well-being (confirmed by Geteri & Angogo, 2013 and Moremi, 2012) which may have contributed to the accumulation of negative emotions such as high anxiety and stress reported in the pre-intervention assessment. Because self-care is the best strategy to overcome emotional exhaustion (Geteri & Angogo, 2013; Wentzel & Brysiewicz, 2014), daily laughter therapy sessions were introduced in this group of care workers.

The results showed that most of the participants of the laughter intervention reported less stress, anxiety and depression after the intervention. Laughter is associated with the cathartic release of accumulated emotions (Goodheart, 1994). Participants had the opportunity to express and release the emotions that they had previously ignored and suppressed. Many of them thus expressed feeling a sense of relief after the intervention. The release of these negative emotions was accompanied by the experience of positive emotions, such as joy, happiness, relief and hope. Fredrickson’s (2001) theory of positive emotions emphasises the process by which individuals’ daily experiences of positive emotions can multiply over time to build an array of substantial personal internal resources. As such, the participants’ exposure to daily laughter therapy sessions facilitated the development of an entire repertoire of positive emotions which can contribute to the alleviation of negative emotional experiences. Laughter induces a form of relaxation that allows people to feel rather than to think. It tends to bypass the cognitive system and focuses on the emotions (Goodheart, 1994). Once positive emotions are activated, it re-energises a person to take a new viewpoint of life and make better judgements. These positive experiences can have a positive cyclic effect on the individual's emotions, cognitions and behaviour, similar to the negative cycle identified by Beck (2011). The positive cycle can be continuously reinforced and broadened through continuous use.

These positive experiences allowed for the development of more positive interactions with co-workers, family members and friends. Positive social interactions again serve to strengthen positive emotions, thereby contributing to the positive cycle of well-being (Vittengl & Holt, 2000; Waugh & Fredrickson, 2006). Many researchers have found that positive social interactions aid in the development and maintenance of psychological well-being (Collins, 2007; Halbesleben, 2006; Medland, Howard-Ruben, & Whitaker, 2007).

The laughter sessions helped the care workers to develop a positive mind set and to take control of their own emotions. This increased their ability to relate and to provide support for the families in their care (Colom et al., 2011). These results contribute to the limited evidence of the potential of laughter as a strategy to reduce stress and counteract negative emotions (Falkenberg, Buchkremer, Bartels, & Wild, 2011; Ko & Youn, 2011).

## Limitations

In this research, qualitative and quantitative data were collected from a small sample of care workers that was selected through purposive sampling, where availability and willingness also played a role. The research mainly focused on the qualitative results obtained from interviews with participants. The quantitative results were used to support the qualitative results. The small sample size made it possible to link the qualitative and quantitative data of each participant to understand their reactions to the intervention. On the other hand, the small sample size and lack of control group data limited the use of the quantitative results. It was not the intention of the research to determine the effectiveness of the intervention – rather to explore the experiences and the value of laughter for the care workers. Due to the nature of qualitative research, the results may have been different if the research was done with another group of care workers. The results may thus not be transferable.

The subjective role of the researcher(s) as in all qualitative research should be noted, although various measures were taken to enhance trustworthy results.

Furthermore, some limitations with the language capabilities of the participants were encountered. Although all the respondents had passed matric through medium English, the participants were not fluent in English which could negatively affected their ability to express their deep-felt emotions.

## Conclusions

There are often media reports on laughter implemented as an intervention in various contexts, but research is seldom done on the experience and value of such sessions for the participants. This research explored the value of laughter as a low-cost intervention for care workers in an HIV context to deal with negative emotions such as anxiety and depression and to promote psychological well-being. Through daily exposure to laughter sessions, the care workers experienced more positive emotions, improved social relationships and improved ways of coping as well as lower levels of anxiety, depression and stress. The triangulation of qualitative and quantitative data made it possible to understand how laughter helped care workers to develop a positive mind set and improved relationships that could improve their ability to provide care for the families they work with.

To improve the services of care workers in South Africa, previous research indicated that they need to be appropriately trained and supported (Dewing et al., 2013; Petersen, Hanass Hancock, Bhana, & Govender, 2014). This research showed that care workers need emotional support and learn self-care skills. When people working in intense emotional situations receive help on a personal level and learn self-care skills, they can develop strengths to cope with stress and compassion fatigue (Meadors & Lamson, 2008) which can contribute to their psychological well-being and care giving. Laughter is a possible resource that is easily available and can be a low-cost strategy to reduce stress and counteract negative emotions among people working in highly emotional environments. This research supports Morreall (1983, 1986) in his unwavering belief that laughter is an important factor in understanding humanity and in contributing to human well-being.
